# Editorial: Advances in surgical management of abdominal and retroperitoneal sarcoma: where do we stand, and where do we go?

**DOI:** 10.3389/fsurg.2024.1488404

**Published:** 2024-09-13

**Authors:** Luit Penninga, Louise Preisler, Jens Georg Hillingsø

**Affiliations:** ^1^ Department of Surgery and Transplantation, Rigshospitalet, Copenhagen University Hospital, Copenhagen, Denmark; ^2^ Department of Clinical Medicine, Copenhagen University, Copenhagen, Denmark

**Keywords:** sarcoma, retroperitoneal tumor, abdominal tumor, evidence based medicine (EBM), surgical approach

**Editorial on the Research Topic**
Advances in surgical management of abdominal and retroperitoneal sarcoma: where do we stand, and where do we go?

The article series on “Advances in Surgical management of abdominal and retroperitoneal sarcoma” describes real-world clinical problems, current challenges, and new management options of sarcomas in these anatomical locations. Examples of real-world clinical problems in the article series are the occurrence of sarcoma types at uncommon locations, and the occurrence of very rare sarcoma types, like primary osteosarcoma of the kidney (Yu et al.), and retroperitoneal undifferentiated pleomorphic sarcoma (Chen et al.). Another frequently-faced problem is a very large tumor-size at presentation. Hence, surgical treatment requires an extensive and major surgical procedure, and sometimes an alternative surgical approach. An example of this in the article series is a thoracotomy for a giant retroperitoneal tumor with diaphragmatic hernia (Hu et al.). In addition, patients happen to present with metastatic disease and new non-surgical treatment options need to be applied. An example of this in the article series is PD-1 inhibitor treatment combined with chemotherapy for metastatic follicular dendritic cell sarcoma of the spleen (Li et al.). Furthermore, a major problem in clinical practice is the very high risk of recurrence after surgical resection of retroperitoneal liposarcoma as reported in two articles in the series (Gao et al., Wang et al.). New treatment strategies are urgently required to reduce the recurrence risk of retroperitoneal sarcomas. These strategies may include more precise surgery, more extensive surgery, (neo)adjuvant chemo and radiation therapy, and other new treatment options. One article in the series reports on the results of preoperative radiotherapy for retroperitoneal liposarcoma, showing that radiotherapy is well-tolerated, though an increase in postoperative blood transfusions and intensive care stay was observed (Jo et al.). However, no effect on local recurrence and survival was observed, which is in accordance with the randomised STRASS-1 trial (1).

The articles series also include systematic reviews on solitary fibrous tumors and leiomyosarcomas (Tolstrup et al., Øines et al.). Prediction of the risk of recurrence in patients with solitary fibrous tumors is a major clinical problem, and proper identification of risk factors for disease recurrence is of utmost importance and summarized in the systematic review on solitary fibrous tumours (Tolstrup et al.). Especially, high mitotic index, Ki67 index and presence of necrosis in surgically resected solitary fibrous tumor increased the risk of recurrence, while TERT promoter mutation appears to be promising component in future risk stratification models (Tolstrup et al.).

The systematic review on abdominal and retroperitoneal leiomyosarcoma in the article series summarizes all available evidence on treatment and diagnosis of these tumors. Of special interest is that the review points out the importance of genetic subtype classification of leiomyosarcomas, as molecular subtype may be more important for tumor behavior and prognosis than tumor location (e.g., abdomen, retroperitoneal, gynecological, extremities) (Øines et al.).

Our article series illustrate the lack of high-quality evidence for the management of abdominal and retroperitoneal sarcoma. There is a great need for well-designed and well-performed prospective studies with relevant clinical and patient reported outcomes. Abdominal and retroperitoneal sarcomas are rare tumors, and special actions are required to establish firm evidence for these seldom cancer types. High-quality evidence can be achieved by performing international multicenter randomised studies. These studies should aim at reducing the risk of recurrence and increase survival. Recent international multicenter RCTs on the effect of neoadjuvant radiotherapy (STRASS-1, completed and published) and neoadjuvant chemotherapy (STRASS-2, currently recruiting) in patients with retroperitoneal sarcomas are excellent examples of how to establish evidence (1–3). In addition, all patients should be registered in national and international clinical registries.

Furthermore, there is a great need for more projects on molecular subtyping and protein expression of different sarcoma tumor types. This will allow for applying individual target treatment approaches. Personalised medicine in sarcoma patients may improve treatment results, reduce recurrence risk and improve survival. An example of this is molecular subtyping for tyrosine kinase inhibitor treatment in patients with gastrointestinal stromal cell tumors (GIST). Personalised medicine will also mean that we can avoid treatments in patients who have no or limited benefits of the treatment. This will reduce adverse treatment effects and improve quality of life. Identification of proper biomarkers may add further to individual-tailored approaches.

Further progress is needed in application of new surgical modalities in sarcoma surgery. Application of fluorescense-guided surgery, irreversible electroporation (Nanoknife®), and microwave ablation are examples of techniques which should be further investigated in the treatment of abdominal and retroperitoneal sarcomas (4–6). Similar to other surgical fields, the benefits and harms of minimal invasive (robotic, laparoscopic and endoscopic) surgery in sarcoma patients should be explored, and Enhanced Recovery After Surgery (ERAS) principles should be fully applied and improved (7, 8). We can conclude that advances are made in the surgical management of abdominal and retroperitoneal sarcoma, though further research is certainly needed to improve outcomes.
